# Creating a sustainable culture of quality through the SLMTA programme in a district hospital laboratory in Kenya

**DOI:** 10.4102/ajlm.v3i2.201

**Published:** 2014-09-16

**Authors:** Phidelis M. Maruti, Ekesa A. Mulianga, Lorna N. Wambani, Melda N. Wafula, Fidelis A. Mambo, Shadrack M. Mutisya, Eric N. Wakaria, Erick M. Mbati, Angela A. Amayo, Jonathan M. Majani, Bryan Nyary, Kilian A. Songwe

**Affiliations:** 1 Ministry of Health-Kenya, Kenya; 2 Department of Health Sciences, Masinde Muliro University, Kenya; 3 A Global Healthcare Public Foundation, Kenya; 4 Management Sciences for Health-Kenya, Kenya; 5 AIDS, Population and Health Integrated Assistance Plus (APHIA PLUS) Western, Kenya; 6 International Healthcare and Development, Nigeria

## Abstract

**Background:**

Bungoma District Hospital Laboratory (BDHL), which supports a 200-bed referral facility, began its Strengthening Laboratory Management Toward Accreditation (SLMTA) journey in 2011 together with eight other laboratories in the second round of SLMTA rollout in Kenya.

**Objectives:**

To describe how the SLMTA programme and enhanced quality interventions changed the culture and management style at BDHL and instilled a quality system designed to sustain progress for years to come.

**Methods:**

SLMTA implementation followed the standard three-workshop series, mentorship site visits and audits. In order to build sustainability of progress, BDHL integrated quality improvement processes into its daily operations. The lab undertook a process of changing its internal culture to align all hospital stakeholders – including upper management, clinicians, laboratory staff and maintenance staff – to the mission of sustainable quality practices at BDHL.

**Results:**

After 16 months in the SLMTA programme, BDHL improved from zero stars (38%) to four stars (89%). Over a period of two to three years, external quality assessment results improved from 47% to 87%; staff punctuality increased from 49% to 82%; clinician complaints decreased from 83% to 16; rejection rates decreased from 12% to 3%; and annual equipment repairs decreased from 40 to 15. Twelve months later the laboratory scored three stars (81%) in an external surveillance audit conducted by Kenya Accreditation Service (KENAS).

**Conclusion:**

Management buy-in, staff participation, use of progress-monitoring tools and feedback systems, as well as incorporation of improvement processes into routine daily activities, were vital in developing and sustaining a culture of quality improvement.

## Introduction

Laboratory systems are one of the core capacities that countries must develop in order to comply with World Health Organization (WHO) International Health Regulations, since they play a major role in the key processes of detection, assessment, response, notification and monitoring of events.^1^ As laboratory results influence up to 70% of medical diagnoses,^2^ reliable laboratory services are essential for the provision of safe and effective treatment to patients. The quality of laboratory services is a major factor that directly affects the quality of healthcare in a country.^2^

The Strengthening Laboratory Management Toward Accreditation (SLMTA) programme promotes rapid, measurable improvement in laboratories of developing countries. SLMTA is implemented through multiple workshops with intervening site visits to support improvement projects.

Kenya began the SLMTA implementation process with 12 laboratories in April 2010. Bungoma District Hospital Laboratory (BDHL) was enrolled in the second SLMTA round in February 2011, along with eight other laboratories. After the first three months of SLMTA implementation, BDHL management noted, from both the internal audit report and general observations, that little progress had been made. As a result, radical changes were phased in to encourage all laboratory staff to participate in improvement activities, adopt more disciplined and stringent work duties and schedules, engage in laboratory planning and include hospital management and other stakeholders in the process. This article describes how the SLMTA programme and enhanced interventions changed the culture and management style at BDHL, instilling a system designed to sustain progress for years to come.

## Research method and design

Bungoma District Hospital, a primary care facility with very limited resources, started operating in 1952 as a Chief’s Native Health Centre. Located in Bungoma Town, it now serves as a referral hospital for the North-western region of Kenya. With 200 in-patient beds, it offers both out-patient and in-patient services and provides laboratory services for haematology, serology, clinical chemistry, immunology, microbiology, parasitology and blood banking.

Consistent with the SLMTA protocol,^3^ a baseline audit was conducted in February 2011, followed by three workshops, two one-week mentorship site visits after each workshop, a mid-term audit and an exit audit in March 2012. To determine the impact of changes made at BDHL and the sustainability of the new systems, an external surveillance audit was conducted by the Kenya Accreditation Service (KENAS) in February 2013, 12 months after SLMTA concluded. The non-profit charity A Global Healthcare Public Foundation played an important role in conducting the three workshops, on-site mentorship and conference call follow up. In addition, the Foundation provided funding for laboratory facility and equipment upgrades.

Efforts were made to engage all hospital management in the process, as well as other stakeholders, including the hospital maintenance unit, the procurement and/or supplies unit and clinicians. SLMTA was integrated by laboratory staff into routine work processes and a succession plan was developed for laboratory management to ensure the sustainability of the quality improvements. This included appointing a deputy for each key function, performing on-the-job mentorship of staff on SLMTA, as well as regular internal reviews of progress. Further engaging stakeholders and ensuring continued progress, the laboratory staff conducted weekly customer surveys (for patients/clients), which informed improvement projects.

SLMTA uses the WHO’s Regional Office for Africa’s (WHO AFRO) Stepwise Laboratory Quality Improvement Process Towards Accreditation (SLIPTA) framework to both guide improvement activities and evaluate programme effectiveness.^4^ Unlike traditional pass/fail accreditation schemes, SLIPTA uses a zero- to five-star scale to recognise the evolving fulfilment of International Organization for Standardization (ISO) 15189 requirements. Laboratories that fail to achieve at least 55% on their audit score receive a zero-star rating; 55% − 64% yields one star, 65% − 74% yields two stars, 75% − 84% yields three stars, 85% − 94% yields four stars and laboratories that achieve 95% or more receive five stars.^4^ This stepwise approach acknowledges laboratories where they stand, supports them with a series of evaluations to demonstrate improvement and both recognises and rewards their progress. The SLIPTA process is not intended to replace established accreditation schemes, but rather to provide an interim pathway to the realisation of international laboratory standards.^5^

Several indicators were measured to assess the impact of SLMTA implementation. Firstly, results from routine External Quality Assessment (EQA) panels from Human Quality Assessment Services (HuQAS), conducted three times per year for 22 analytes, were compared; the average annual percentage of correct responses from 2010 to 2013 are presented. Secondly, staff punctuality in 2011–2013 was assessed based on data from an employee time clock, defined as the average overall percentage of person-days that staff arrived on time for their shift. Thirdly, clinician and customer satisfaction were assessed by means of a ‘How do you rate us’ form that was made available to all patients in 2012–2013 and clinicians in 2011–2013; the proportion of forms submitted with complaints was calculated. Fourthly, annual average sample rejection rates for all laboratory tests were calculated for 2011–2013; and finally, equipment repairs and the proportion carried out by external engineers versus internal staff from the hospital’s biomedical engineering department were assessed for 2011–2013.

## Results and discussion

**FIGURE 1:**
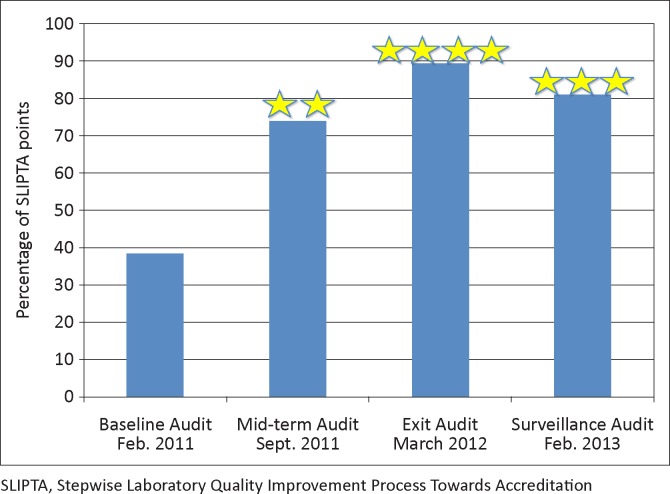
Results for baseline, mid-term, exit and surveillance audits, Bungoma District Hospital Laboratory, Kenya.

BDHL’s baseline audit score was 38% (0 stars). After SLMTA implementation, the laboratory scored 89% (4 stars) at the exit audit. The surveillance audit carried out 12 months afterward yielded a score of 81% (3 stars) (Figure 1).

Without hospital management support, sustainable changes are difficult to achieve. Poor EQA data for chemistry and haematology, as well as an increased mortality rate in medical wards from 3% in 2009 to 9% in 2011, were presented to management to demonstrate that laboratory failures could be contributing to deaths, especially amongst HIV patients for whom treatment depends heavily on chemistry results. Hospital management approved the purchase of a new fully-automated analyser to replace the old semi-automated analyser and control materials, and convinced partners to donate air conditioners for the laboratory. As a result, erratic temperatures no longer interfered with the quality of results or turnaround time and overall EQA results improved from 47% in 2010 to 87% in 2013 – above the set target of 80% (Table 1).

**TABLE 1:** Summary of quality indicators before and after SLMTA implementation.

Indicator	2010 %	2011 %	2012 %	2013 %
EQA results†	47	78	86	87
Staff punctuality	N/A	49	77	82
Clinician complaints‡	N/A	83	28	16
Customer complaints*	N/A	N/A	3	22
Sample rejection rate	N/A	12	4	3
Number of equipment repairs	N/A	40	10	15
Proportion of repairs by external engineer	N/A	80	30	20

SLMTA, Strengthening Laboratory Management Toward Accreditation.

† EQA, external quality assessment, based on 66 test panels per year; N/A, not available;

‡ Based on 76 submitted forms in 2011, 65 in 2012 and 252 in 2013;

* Based on 204 submitted forms in 2012 and 85 in 2013.

Because staff buy-in is also crucial, further efforts were made to encourage participation throughout the hospital. An annual award scheme for the entire hospital was established in order to motivate staff to improve patient care. In addition, a ‘Wall of Fame’ and a ‘Wall of Shame’ were instituted in order to further inculcate a culture of friendly competition amongst staff and to ensure conformity to the set standards. These activities led to the prompt release of test results, thereby improving turnaround time and the efficiency and quality of patient care throughout the hospital. However, the laboratory experienced substantial staff turn-over in 2013, with four laboratory personnel being transferred, including the medical superintendent and laboratory manager. This left the laboratory with nine staff members, thus causing acute staff shortages and gaps in laboratory management, with the result being that customer complaints increased from 3% in 2012 to 22% in 2013. A new policy for clocking-in and -out for laboratory staff and the introduction of a leave request form resulted in an increase in staff punctuality from 49% in 2011 to 82% in 2013 (Table 2), as well as an increase in staff availability to perform assigned tasks. This temporarily addressed staff shortages.

**TABLE 2:** Summary of improvement activities.

Improvement activities	Purpose	Outcome
Total hospital management	To obtain hospital support and provide a link with other hospital departments	Financial support and total hospital team involvement
Meeting with procurement and maintenance units	To ensure proper supplies and periodic equipment maintenance	Availability of supplies with required specifications and reduction of equipment down time
Laboratory–clinician meetings	To provide link between laboratory and clinical department	Reduction in complaints and sample rejection rates; increase in clinician confidence in the laboratory
1-on-1 mentorship	To remind staff of laboratory objectives and address individual weaknesses	All staff focused on quality improvement
Time management	To improve staff punctuality	Staff available to perform assigned tasks
‘Wall of Fame’ and ‘Wall of Shame’	To improve staff attitude and motivation	Competition amongst staff to improve quality
Succession plan	To ensure continuity of improvement process	Continuous improvement even in the absence of key personnel
Customer survey	To solicit customer feedback to guide improvement	Feedback used for further improvement of the laboratory process
Internal audits	To monitor progress	Gaps identified and addressed
External surveillance audit	To track sustainability of improvement	Evidence of continued quality management in the absence of mentorship

Quarterly meetings for one-on-one mentorship with each laboratory staff member were introduced. During these meetings, laboratory management reminded staff members of strategic objectives, thanked them for their hard work, provided feedback on their performance and suggested areas of improvement. In response to positive feedback and this collaborative approach, laboratory staff members reported feeling appreciated, more engaged and willing to be part of a team to improve healthcare quality in the hospital.

The laboratory also began holding quarterly meetings with clinicians to discuss sample rejection rates, clinicians’ perception of the laboratory and suggestions for improvement. The proportion of clinicians who reported complaints on the feedback form decreased from 83% in 2011 to 16% in 2013, whilst the total number of form submissions increased from 76 to 252. Sample rejection rates declined from 12% in 2011 to 3% in 2013 and clinicians reported in meetings that their confidence in laboratory results had improved. The laboratory also met regularly with stakeholders from the maintenance and procurement departments in order to advocate for prompt routine preventive equipment maintenance. Consequently, the number of equipment repairs decreased from 40 in 2011 to 15 in 2013 and the proportion of repairs conducted by an external engineer versus the hospital biomedical engineering department decreased from 80% to 20% (Table 1).

In order to sustain the gains achieved, SLMTA was integrated into daily routines, building a foundation for continuous improvement. Discussions of improvement projects are now included in regular laboratory staff meetings; the laboratory conducts weekly hands-on continuous medical education sessions; and all staff members are now involved in budget and planning discussions. These changes were designed in order to improve the laboratory staff’s customs, beliefs and attitudes in the workplace, leading to widespread and lasting staff support of laboratory quality improvement activities.

Sustainability is further enhanced by quarterly internal audits, conducted by the laboratory quality officer. To monitor processes, staff members identify causes of problems and suggest possible solutions. A root cause analysis is conducted and corrective action is identified, following a two-step procedure.

**FIGURE 2:**
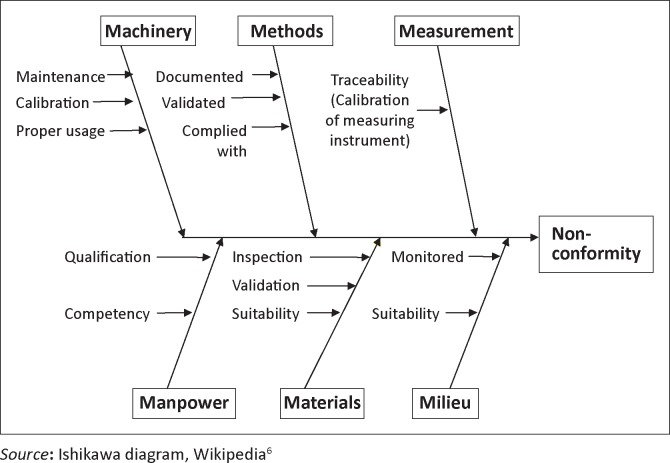
Cause-and-effect diagram.

Step 1 involves the development of a Cause-and-Effect Diagram (Figure 2) in order to categorise probable causes of non-conformity under ‘the 6 Ms’ of Machinery, Methods, Measurement, Manpower, Materials and Milieu (environment).^6^ Using objective evidence, the quality officer then examines each probable cause and, based on the evaluation, the staff then works by process of elimination to identify those items which were most likely associated with the non-conformity.

Step 2 is the root cause investigation. Using the problems identified in Step 1, an investigation into their root cause is performed. For example, the quality officer may recognise that laboratory personnel do not have proper competency records. To find the source of this problem, the officer may ask ‘Why?’ and then receive a variety of answers, including:

- Staff are not aware of the need for competency assessment.
- Staff were not trained on the procedure for competency assessment.
- The Quality Officer thought the procedure was covered during training.
- No records of training are kept.
- The training procedure does not mention the need to keep records.

After this questioning, the root cause of the problem may become clear: for example, perhaps the training procedure does not fully address the need for record keeping. The quality officer may then recommend that, in order to improve the system, the training procedure must be revised. This process of identifying problems and selecting improvement activities allows for a clear understanding of what is hindering efficient and reliable work in the laboratory and provides appropriate solutions for improvement (Table 2).

### Conclusion

BDHL successfully used SLMTA to progress from zero to four stars within a 16-month period and to maintain a three-star rating for 12 months thereafter. This quality improvement required substantial effort and a collaborative approach. Fundamental steps were necessary in order to create and maintain a culture that supports continuous quality improvement. Firstly, universal rules were established and enforced, such as adopting written protocols and practices that prescribe clear policies, procedures, values and behaviours. Secondly, the principles and techniques of quality improvement and their associated behaviours were taught so that staff members could learn both the concepts and how to apply them. Finally, it was critical to reinforce these principles and behaviours on a continual basis. BDHL employees were recognised and rewarded when they demonstrated adherence and consequences were made clear for noncompliance. Leaders and managers did not allow laboratory staff to become complacent, simply meeting basic or minimum requirements. Everyone was pressed continually for professional excellence, growth and improvement.

For quality management systems to be implemented effectively and sustained, the hospital’s management and staff must be involved and willing to participate. BDHL can attest that sustainable improvement is achieved by engaging all hospital stakeholders, leading to increased confidence in the laboratory on the part of clinicians, nurses and patients. Long-term sustainability will rely on continued vigilance, training of new staff and participation of all stakeholders.
